# Descemet Stripping Only for Symptomatic Fuchs Endothelial Dystrophy—A Retrospective Case Series Comparing ROCK-I vs. Hypertonic Sodium Chloride for Post-Surgical Adjuvant Therapy

**DOI:** 10.3390/jcm14051512

**Published:** 2025-02-24

**Authors:** Eyal Cohen, Nizar Din, Sultan Aldrees, Michael Mimouni, Tanya Trinh, Nir Sorkin, Larissa Gouvea, Clara C. Chan, Allan R. Slomovic

**Affiliations:** 1 Department of Ophthalmology and Vision Sciences, University of Toronto, Toronto, ON M5T 2S8, Canada; nizar.din@uhn.ca (N.D.); sultan.aldrees@mail.mcgill.ca (S.A.); michael@intername.co.il (M.M.); tanya.trinh@gmail.com (T.T.); gouveaalarissa@gmail.com (L.G.); clara.chan@uhn.ca (C.C.C.); allan.slomovic@utoronto.ca (A.R.S.); 2 Department of Ophthalmology, Tel Aviv Medical Center, Sackler Faculty of Medicine, Tel Aviv University, Tel Aviv 6997801, Israel; nir_sorkin@yahoo.com; 3 Department of Ophthalmology, Rambam Health Care Campus, Haifa 3109601, Israel

**Keywords:** Descemet stripping only, Descemetorhexis without endothelial keratoplasty, Descemetorhexis, Fuchs corneal endothelial dystrophy, two flaps technique

## Abstract

**Purpose**: To report our experience with Descemet stripping only (DSO) for the treatment of Fuchs endothelial corneal dystrophy. **Methods:** Thirteen eyes of 9 patients with symptomatic Fuchs endothelial dystrophy underwent a 4 mm central Descemetorhexis without graft implantation between June 2017 and July 2020. All patients had central confluent guttata, undetectable central endothelial cell count by specular microscopy, and healthy peripheral corneal endothelium. In 6 eyes, the procedure was combined with phacoemulsification and intraocular lens implantation. Eight eyes were treated with topical rho-associated protein kinase (Rock) inhibitors and five eyes were treated with hypertonic sodium chloride 5%, post operatively. **Results:** All eyes completed at least 4 months of post-operative follow-up (mean follow-up 12.0 ± 7.9 mo; 4–29 mo). Mean patient age was 70 ± 6 years. All eyes achieved corneal clearance with an average time for clearance of 7.2 ± 2.4 weeks. Mean endothelial cell count postoperatively was 778 ± 228. Mean central corneal thicknesses pre- and postoperatively were 620 ± 100 and 560 ± 58 μm, respectively. Eleven eyes achieved improvement in visual acuity and in two eyes vision remained unchanged, with mean visual acuity 0.392 to 0.225 logMAR; *p* = 0.001. Also, all patients reported subjective improvement in the quality of vision. ROCK inhibitors compared to hypertonic sodium chloride 5% did not show statistically significant differences in time for corneal clearance or endothelial cell counts postoperatively but did show a trend towards faster corneal clearance and higher endothelial cell counts postoperatively among the ROCK inhibitors-treated eyes. **Conclusions:** In patients with Fuchs endothelial dystrophy and visual degradation secondary to central guttata, DSO represents a viable procedure for visual rehabilitation.

## 1. Introduction

The surgical management of Fuchs endothelial corneal dystrophy (FECD) has advanced rapidly in the past two decades. The introduction of endolamellar keratoplasty, in the form of Descemet stripping endothelial keratoplasty (DSEK) and Descemet membrane endothelial keratoplasty (DMEK), has significantly improved corneal grafting outcomes for patients affected by FECD [1,2]. Endothelial keratoplasty provides faster visual recovery, less intraoperative complications, reduced postoperative astigmatism, and lower rejection rates compared with penetrating keratoplasty [3]. However, endothelial keratoplasty still carries the risk of graft failure secondary to surgical complications (such as graft dislocation), primary graft failure, graft rejection [4], and a steep learning curve is still present for the novice surgeon [5]. Endothelial keratoplasty is also limited by tissue availability worldwide [6].

Anecdotal case reports have described spontaneous corneal clearance after accidental trauma to the Descemet–endothelial complex during cataract surgeries. Furthermore, other case reports have demonstrated corneal clearance despite DMEK graft detachment in FECD [7,8,9].

These findings have suggested the potential for endothelial “rejuvenation” and cell migration and calls into question the need for donor endothelial transplantation in the setting of corneal decompensation in all patients with Fuchs corneal endothelial dystrophy.

Several corneal centers around the world have attempted to exploit this potential endothelial migration by performing a planned surgical central Descemetorhexis without donor endothelial transplantation in FECD patients. Early reports of success were mixed at best [10,11,12]. However, recent studies with careful patient selection and enhanced surgical techniques, combined with the use of postoperative topical ROCK inhibitors to aid endothelial migration and healing [13], have shown promising results with corneal clearance ranging from 63 to 100% [14,15,16].

The purpose of this study is to investigate the efficacy of Descemet stripping only (DSO) for the management of symptomatic FECD and to describe preliminary surgical results comparing between the use of ROCK inhibitors and hypertonic sodium chloride 5% as adjuvant treatment postoperatively.

## 2. Methods

This retrospective institutional observational study was conducted in compliance with the tenets of the Declaration of Helsinki and received Research Ethics Board approval from University Health Network (Toronto Western Hospital, Toronto, ON, Canada).

### 2.1. Study Participants

A retrospective chart review was performed, including all eyes with symptomatic FECD who underwent Descemet stripping only (DSO) with or without phacoemulsification and intraocular lens implantation (IOL) between June 2017 and August 2020, at the Toronto Western Hospital and the Kensington Eye Institute (Toronto, ON, Canada). All included eyes had completed at least 4 months follow-up postoperatively. All surgeries were performed by two experienced corneal surgeons (A.R.S., C.C.) or by corneal fellows under their direct supervision. A detailed patient consultation regarding the pros and cons of DSO compared to lamellar corneal transplantation was given to the patients and a rescue lamellar corneal transplant was offered to the patients in case the cornea would not recover.

### 2.2. Data Collection

Data collected in this study included patients demographics, best spectacle-corrected visual acuity (BSCVA), slit lamp clinical findings including dilated fundus examination, corneal topography with corneal astigmatism (OPD Scan II ARK 10000, NIDEK, Tokyo, Japan), endothelial cell count, and central corneal pachymetry using non-contact specular microscopy (Robo, KSS 300; Konan Medical, Hyogo, Japan). Intraoperative and postoperative complications, postoperative treatment given, and time for corneal clearance postoperatively were also collected.

### 2.3. Surgical Technique

When DSO was performed alongside cataract extraction, an 8-point ink-marked caliper is applied on the corneal epithelial surface, sized at 4 mm diameter overlying the non-dilated pupil. Then, 0.4 mL of phenylephrine 2.5%, diluted in a 1:3 ratio with balance salt solution, and 0.4 mL of 1% lidocaine was injected intracamerally to promote pupil dilatation and provide additional anesthesia, respectively. After cataract extraction and intraocular lens implantation and before removing the viscoelastic, the Descemet–endothelial complex was stripped using the Gorovoy forceps. In ten of our cases the surgical “two-Flaps” technique previously published by our group [17] was used with an initial Descemet flap created in an anti-clockwise manner for two clock hours followed by continuation of this flap in the opposite clockwise direction for two clock hours. This creates a four-clock hour opening in the Descemet membrane, which is initially pulled centrally to provide slack before completion of a continuous curvilinear Descemetorrhexis. However, in three of our cases (carried out by one surgeon, CC), a reverse Sinskey hook was used to score initially 2 clock hours, followed by Utrata forceps to complete the Descemetorrhexis set at the central 4mm. Viscoelastic is then removed by irrigation/aspiration and the main wound is hydrated. When performed as DSO alone, all steps were identical to the above except for the cataract extraction part.

### 2.4. Postoperative Management

All patients were examined one day post-surgery and were started on the combined 0.1% dexamethasone sodium phosphate and 0.3% tobramycin antibiotic (Tobradex; Alcon, Mississauga, ON, Canada) eye drops 4 times daily for one week. The Tobradex was then discontinued and 0.1% dexamethasone sodium phosphate (Maxidex; Alcon Labs Inc., Fort Worth, TX, USA) was started 4 times daily and was tapered down over a four-week period. ROCK inhibitors, ripasudil hydrochloride hydrate (Glanatec ophthalmic solution 0.4%, Kowa Co Ltd., Nagoya, Japan), four times daily, were used for eight eyes for at least 2 months. When corneal clearance was not achieved after two months, the patient was instructed to continue ripasudil four times daily and was closely monitored monthly until corneal clearance was achieved. Five eyes that did not receive ripasudil (due to accessibility issues) were treated with hypertonic sodium chloride 5% (Muro 128, Bausch&Lomb, Bridgewater, NJ, USA) four times daily until cornea clearance was achieved. Patients were re-examined at 1 week, 1 month, 2 months, every 3 months in the first postoperative year, every 6 months in the second postoperative year, and annually thereafter.

### 2.5. Study Outcomes

Primary study outcomes included rate and time for corneal clearance. Secondary outcomes included rate of intraoperative and postoperative complications, BSCVA, endothelial cell density, and central corneal pachymetry at last follow-up. Further subgroup analysis was performed to assess the impact and influence of postoperative ROCK inhibitors compared to sodium chloride 5% (Muro 128, Bausch&Lomb, Bridgewater, NJ, USA) on time for corneal clearance and postoperative ECD.

### 2.6. Statistical Analysis

Data were entered in Microsoft Excel (2019)™ and analyzed using Minitab Software, version 19 (Minitab Inc, State College, PA, USA). For the analysis of paired continuous data, the paired student’s *t*-test or Wilcoxon test were used wherever appropriate. For un-paired continuous data, the Kruskal–Wallis test was used. For the analysis of categorical variables, the chi-square test or Fisher exact test were used wherever appropriate. In all analyses a two-sided *p* value < 0.05 was considered statistically significant. All presented means are accompanied by their respective standard deviations.

## 3. Results

Thirteen eyes of nine patients underwent DSO for the treatment of symptomatic FECD. All eyes had central corneal guttata with some degree of central corneal edema but corneal peripheries were clear without a prominent peripheral failure.

Six eyes (46%) had a combined procedure with phacoemulsification and IOL implantation, with all eyes having at least 4 months of follow-up, with a mean follow-up of 12.0 ± 7.9 months.

The mean age was 70 ± 6 years and 69% (n = 9) were female. Preoperative BSCVA was 0.392 ± 0.27 LogMAR (Snellen equivalent 20/50) and postoperative mean BSCVA was 0.225 ± 0.26 LogMAR (Snellen equivalent 20/30); *p* = 0.001. Vision improved in all cases except for two patients whose vision remained unchanged. Three eyes had reliable preoperative ECD with an average of 485 cells/mm^2^, however, in ten eyes reliable ECD readings could not be obtained. Mean preoperative central corneal thickness (CCT) was 620 ± 100 μm. Table 1 summarizes demographics and the preoperative data.

Postoperatively, the mean ECD on last follow-up was 778 ± 228 (range 508 to 1176) cells/mm^2^, and the mean CCT was 560 ± 58 μm.

No intraoperative complications were recorded. Postoperatively, deep stromal nodular scars, correlated to the area where Descemetorhexis was initiated, was documented in three eyes but tended to fade in subsequent follow-up visits and was not visually significant. One eye, with known preoperative epi-retinal membrane (ERM), which had DSO only, developed mild cystoid macular edema postoperatively. Topical treatment with Nepafenac 0.1% (Nevanac; Alcon Labs, Fort Worth, TX, USA) three time daily and prednisolone acetate 1% (Pred-Forte ophthalmic suspension; Allergan, Inc., Irvine, CA, USA) four times daily was applied for two months without complete resolution; the patient was referred for retinal consult to consider ERM peeling.

All corneas cleared on clinical exam with a mean time for corneal clearance of 7.2 ± 2.4 (range 5 to 12) weeks. Eight eyes and five eyes were treated with ROCK inhibitors and Muro 128, respectively, postoperatively. The time for corneal clearance did not differ significantly between the groups (6.93 ± 2.4 weeks versus 7.60 ± 2.6 weeks, respectively, *p* = 0.417). Similarly, there was no statistical difference in the ECD between both groups (916 ± 225 cells/mm^2^ versus 697 ± 208 cells/mm^2^, respectively, *p* = 0.297). Table 2 summarizes the postoperative data.

Four patients had bilateral surgery; in all cases both eyes of the same patient had similar times for corneal clearance and visual recovery with differences between the two eyes not exceeding one week.

Six eyes had combined phacoemulsification, intraocular lens implantation, and DSO; all eyes were targeted to slight myopic spherical equivalent (SE) results, with a mean targeted SE of −0.65 (range −0.1 to −1.0) diopters. Final SE was recorded for four eyes, with a mean final SE of −0.47 diopters; all four eyes achieved final SE results within ±0.5 diopter from the SE target.

Seven eyes had DSO as a single procedure; all of them had preoperative visual disturbances like glares, halos, diurnal visual fluctuation, and a degree of corneal edema with a mean CCT of 647 ± 152 μm and mean visual acuity of 0.33 ± 0.19 LogMAR preoperatively. At last follow-up all of these eyes had visual symptomatic improvement with mean visual acuity of 0.20 ± 0.22 LogMAR and a mean CCT of 538 ± 61 μm.

## 4. Discussion

In our cohort of patients who had DSO, all 13 eyes showed corneal clearance postoperatively. Eleven eyes demonstrated visual improvement postoperatively whilst two eyes had unchanged vision throughout follow-up. Of those two, one developed mild cystoid macular edema which did not improve under topical treatment and was referred to a retina specialist, and one eye regained vision of 20/30 at 4 months follow-up without further improvement.

Previous studies have shown that ROCK inhibitors may fasten corneal clearance. The mechanisms by which ROCK inhibitors facilitate these outcomes include promoting endothelial cell proliferation, migration, and wound healing, as evidenced by both in vivo and ex vivo studies [18,19]. These inhibitors also help maintain the integrity of the endothelial barrier and reduce corneal edema, which is crucial in the postoperative management of DSO patients. Macsai et al. in their series showed a quicker visual recovery in the ripasudil group compared to the untreated group (4.6 vs. 6.5 weeks, *p* < 0.01) [20]. Ripasudil treatment is also considered to act as a salvage treatment for non-clearing corneas post DSO. Moloney and his group described three cases with persistent corneal edema for at least 2 months post-DSO. These patients were treated with ripasudil six times per day and corneal clearance was achieved by two weeks [13].

In our study, when we were unable to provide ROCK inhibitors as an adjuvant post-surgical therapy, we administered hypertonic sodium chloride as an adjuvant post-surgical treatment, considering that hypertonic saline could facilitate endothelial function by drawing fluids from the cornea and reducing the functional load on the remaining active endothelial cells. This approach is similar to the standard practice of using hypertonic saline in cases of corneal edema following cataract surgery with delayed corneal recovery.

In our cohort, ROCK-I did not play a significant role for fastening corneal clearance compared to hypertonic sodium chloride 5%.

ROCK inhibitors have also been proposed to improve ECD post operatively after DSO. This was described by Macsai et al., who reported higher ECD in the treated eyes compared to the non-treated eyes [20]. Similarly, netarsudil has been shown to significantly reduce the time to corneal clearance, improve best-corrected visual acuity, and increase central endothelial cell count at 6 and 12 months postoperatively [21].

In our cohort, the mean ECD was higher for the ROCK inhibitor group compared to the sodium chloride group, 916 ± 225 vs. 697 ± 208 cells/mm^2^, respectively, but did not reach statistical significance. We assume that a larger sample size may reach significance.

In our series, three eyes developed postoperative deep nodular stromal scars at the area where Descemetorehxis was initiated. Two of these cases were performed using a Sinskey hook and one with the two flaps technique described above. It has been observed that a postoperative deep stromal scar usually corresponds to the location where the scoring has taken place [22]. The theory, therefore, is that the surgical trauma induced by the instrument used for performing Descemet scoring initiates an inflammatory, pro-scarring response that leads to stromal keratocyte activation, proliferation, and fibrosis [22]. Furthermore, specular microscopic analysis has shown the appearance of a stromal trench at the site of initiating the scoring process. This can act as a barrier impeding endothelial cell migration from the periphery into the central peeled zone [22]. The “Two flaps” technique described above uses the non-sharp surface of the Gorovoy forceps for creating the first opening in Descemet membrane and then continues by peeling two flaps of 2 clock hours each for both sides of the first opening. With a 4-clock hour opening in Descemet membrane the peeling is continued with a continuous curvilinear peeling technique. This helps reduce trauma to the underlying stroma and thereby reduces the risks of stromal scarring and stromal trench formation.

Patient selection may also have a role in DSO outcomes but requires further investigation. Factors such as age, sex, systemic diseases such as diabetes mellitus, smoking history, or genetic factors have not been thoroughly investigated; however, ex vivo human corneal culture models have shown that the presence of an intact Descemet membrane, young donor age, and supplementation with the Rho kinase inhibitor Y-27632 promotes corneal endothelial cell migration [23].

Four patients had bilateral surgery, and the time for corneal clearance was similar between both eyes and did not exceed one week between the two eyes. This finding is in keeping with previous literature findings [22,24], suggesting that other factors such as growth factors in the anterior chamber or genetic factors may also influence the rate of endothelial cell migration and corneal clearance.

There are several limitations to this study, including its retrospective nature, small sample size, and inconsistent follow-up due to the latest pandemic of the Corona virus disease 19. Furthermore, endothelial cell density measurements were captured by specular microscopy and peripheral cell counts were difficult to obtain. Utilizing confocal microscopy could help better understand the role of peripheral cell counts as a factor for faster recovery and surgical outcomes. However, in our group all patients had no clinically obvious guttata extending beyond the central 6 mm.

In conclusion, according to our study, Descemet stripping only seems to be a valid surgical option for treating symptomatic FECD patients without significant corneal decompensation if guttate is located centrally. Our preliminary results comparing between postoperative use of ROCK inhibitors and Muro 128 did not show statistically significant differences in time for corneal clearance or endothelial cell counts postoperatively but showed a trend towards faster healing and higher endothelial cell counts among the ROCK inhibitors-treated group. Further studies with larger sample sizes are warranted to compare between these groups and finding additional surgical and environmental factors associated with DSO surgical success.

## Figures and Tables

**Table 1:** Patient demographics, and preoperative and operative data.

Mean Patient Age, Years (SD)	70 (6)
Female gender, n (%)	9 (69%)
Laterality: left eyes (%)	7 (53%)
Lens status: phakic, n (%)	8 (62%)
Combined phacoemulsification and DSO Surgery, n (%)	6 (46%)
Mean BSCVA, log MAR (SD)	0.392 (0.27)
Mean central corneal thickness, μm (SD)	620 (100)
* Mean ECD, cells/mm^2^ (SD)	485 (61)

* Availability: 3 eyes; BSCVA: best spectacle-corrected visual acuity; DSO: Descemet stripping only; ECD: endothelial cell density.

**Table 2:** Postoperative data.

	Entire Cohort	ROCK-I	Hypertonic Sodium Chloride 5%	*p*-Value
Mean time of follow-up, months (SD)	12.0 (7.9)	8.58 (4.47)	17.71 (9.55)	0.067
Corneal clearance rate, n (%)	13 (100%)	8 (100%)	5 (100%)	
Mean time for corneal clearance, weeks (SD)	7.2 (2.4)	6.93 ± 2.4	7.60 ± 2.6	0.417
* Mean BSCVA, log MAR (SD)	0.225 (0.26)	0.234 (0.27)	0.212 (0.27)	0.608
* Mean ECD, cells/mm^2^ (SD)	778 (228)	916 ± 225	697 ± 208	0.297
* Mean central corneal thickness, μm (SD)	560 (58)	571 (50)	544 (73)	0.286

* As measured at last follow-up visit; BSCVA: best spectacle-corrected visual acuity; DSO: Descemet stripping only; ECD: endothelial cell density; ROCK-I: rho-associated protein kinase inhibitors.

## Data Availability

Research data will be available upon request.
